# Graph Neural Networks and Structural Information on Ionic Liquids: A Cheminformatics Study on Molecular Physicochemical Property Prediction

**DOI:** 10.1021/acs.jpcb.3c05521

**Published:** 2023-11-28

**Authors:** Karol Baran, Adam Kloskowski

**Affiliations:** Department of Physical Chemistry, Faculty of Chemistry, Gdansk University of Technology, Narutowicza Street 11/12, 80-233 Gdansk, Poland

## Abstract

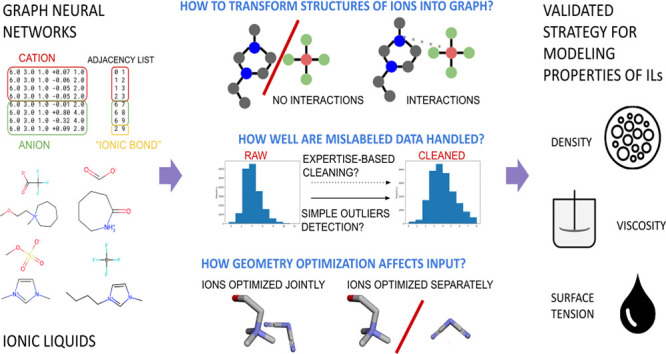

Ionic liquids (ILs) provide a promising solution in many industrial applications, such as solvents, absorbents, electrolytes, catalysts, lubricants, and many others. However, due to the enormous variety of their structures, uncovering or designing those with optimal attributes requires expensive and exhaustive simulations and experiments. For these reasons, searching for an efficient theoretical tool for finding the relationship between the IL structure and properties has been the subject of many research studies. Recently, special attention has been paid to machine learning tools, especially multilayer perceptron and convolutional neural networks, among many other algorithms in the field of artificial neural networks. For the latter, graph neural networks (GNNs) seem to be a powerful cheminformatic tool yet not well enough studied for dual molecular systems such as ILs. In this work, the usage of GNNs in structure–property studies is critically evaluated for predicting the density, viscosity, and surface tension of ILs. The problem of data availability and integrity is discussed to show how well GNNs deal with mislabeled chemical data. Providing more training data is proven to be more important than ensuring that they are immaculate. Great attention is paid to how GNNs process different ions to give graph transformations and electrostatic information. Clues on how GNNs should be applied to predict the properties of ILs are provided. Differences, especially regarding handling mislabeled data, favoring the use of GNNs over classical quantitative structure–property models are discussed.

## Introduction

Ionic liquids (ILs) are organic salts that exist in a liquid state at or near room temperature.^1^ They have been widely studied due to their unique properties, such as nonvolatility, wide electrochemical window, high thermal stability, and tunability. The ionic nature and tunability of ILs allow them to be designed and tailored for specific uses.^2^ By choosing suitable cation and anion combinations, ILs can be engineered to possess appropriate solvation properties,^3^ viscosity,^4^ melting point,^5^ density,^6^ and other physicochemical characteristics for a particular application.^7^ This gives ILs the title “designer solvents”. The structure–property relationships of ILs provide guidelines for the rational design of novel ILs with targeted performance as designer solvents.^8^

The molecular structure of chemical compounds, including ILs, is the fundamental determinant of their properties, as different arrangements of atoms within the molecule lead to varying chemical (both inter- and intramolecular) interactions, which define the compound’s behavior and characteristics. Understanding the relationships between molecular structure and properties is important for rational materials design and discovery. Quantitative structure–property relationship (QSPR) studies aim to model these associations^9^ mathematically. The common approach is to represent molecules via a set of molecular descriptors or depiction of how many times predefined groups contribute to the formation of a molecule. QSPR modeling has several advantages over traditional experimental methods, such as experimental screening or molecular simulations. It is relatively fast and inexpensive compared to the synthesis and characterization of large numbers of compounds.^9^ QSPR models also provide insights into the key molecular features influencing a particular property. This understanding can guide the development of new chemical entities with targeted properties. Furthermore, once developed, QSPR models can be used to predict the properties of untested compounds by using proper mapping of molecular features describing the structure of a compound with a target property.^10^

Multiple linear regression, popular in QSPR, might not capture nonlinear relationships present in complex and multidimensional chemical space.^11^ Linear models are popular and easy to build and operate on molecular descriptors. However, there are issues arising in the interpretation and selection of important features.^12^ In machine learning (ML), there are many possible algorithms to deal with that kind of issue, among which random forest,^13^ gradient boosting,^14^ and simple multilayer perceptron^15^ should be listed. However, algorithms that allow capturing the most complex relations are neural networks (NN).^16^ The NNs were successfully applied to predict the density,^17^ viscosity,^18^ and toxicity^19^ of ILs. Besides the algorithm itself, data set size also seems to be an important limitation in QSPR studies. Data set length may vary from about 30 samples^20^ to as much as 50,000 or more.^21^ Its size might play a critical role in the model’s overall performance.^22^ A separate issue is the quality of the data set used for training the model as well as for its validation and testing. In the literature, set cleaning applies to both the chemical correctness of structures^23^ and the values of output variables.^24^

Graph neural networks (GNNs) have emerged as a promising tool for molecular studies due to their ability to handle graph-structured data. Their application potential results from the way of representing the molecular structure, where molecules are represented as graphs where atoms and their related properties are encoded in the form of nodes, while chemical bonds are in the form of connections in the graph. GNNs are a type of deep neural network that can learn representations of graph-structured data by recursively aggregating and transforming information from local neighborhoods of nodes. Compared with traditional ML methods that assume data are represented as vectors or matrices, GNNs can directly process molecular graphs, which are essential for studying chemical compounds and their properties. GNNs represent a significant step forward in the development of deep neural networks for molecular studies and have the potential to enable more accurate predictions and discoveries in drug discovery and material science. However, they account for only a limited number of studies on molecular property prediction (MPP).^25^ Recently, the use of GNNs in many fields of chemistry is under investigation.^23^ In the case of predicting properties of ILs, GNNs were applied to predict the solubility of carbon dioxide^24^ and activity coefficients of solutes important from the environmental perspective.^26^ The two studies confirmed that GNNs are well-suited for structure–property studies. GNNs excel in capturing complex structural relationships within molecular graphs, which is crucial for ILs’ property prediction due to the intricate dependencies among molecular constituents and the complex nature of dual molecular systems. By harnessing GNNs, researchers can potentially achieve superior predictive accuracy and gain deeper insight into the structure–property relationships of ILs, thereby advancing their tailored design for various applications. However, there are some open questions that were not addressed in these studies, mostly regarding comparison of different graph formation possibilities used and data quality needs of the GNN algorithm. Issues regarding outlier detection are also interesting to be covered. Modelability analysis is becoming interesting issue in the field of MPP, and it is proposed that one should be aware of algorithmic ways of detecting outliers, as well as points being close to hyperplanes separating datasubsets.^27^ Finally, transfer learning as a tool that might increase models’ performance is heavily investigated using various deep^28^ and convolutional^29^ neural networks.

Therefore, it would be interesting to obtain more in-depth insight into how GNNs process ILs that are specific compounds, the properties of which depend not only on the structure of subcomponents (ions) but also on their combination. The study used data on the physicochemical properties of ILs, well described and documented in the literature, i.e., density, viscosity, and surface tension. The data sets used also cover the dependencies of the mentioned properties on the conditions of measurement (temperature and pressure). Several key issues are detailed, analyzed, and discussed. First, the impact of data set size and outlier detection methods is established. Second, the impact of different ways of representing IL molecules as graphs and how electrostatic information should be provided to GNNs are examined. Finally, the possibility of transfer learning and fine-tuning is examined. In the literature on ILs, the topics addressed are not widely studied, and the authors would rather use one of the tested strategies without in-depth validation of other options.^26^ Obtaining answers to those questions allows for obtaining a well-performing model for structure–property modeling of ILs properties using GNNs in a systematic approach. Our main goal is to gain some insights into how GNNs handle chemical structural information for complex chemicals such as ILs (that might be treated as a mixture of two different species). Finally, the model performance and model-building procedure are critically compared with previously reported findings in the source article.

## Methods

### Research Problems

**Figure 1:**
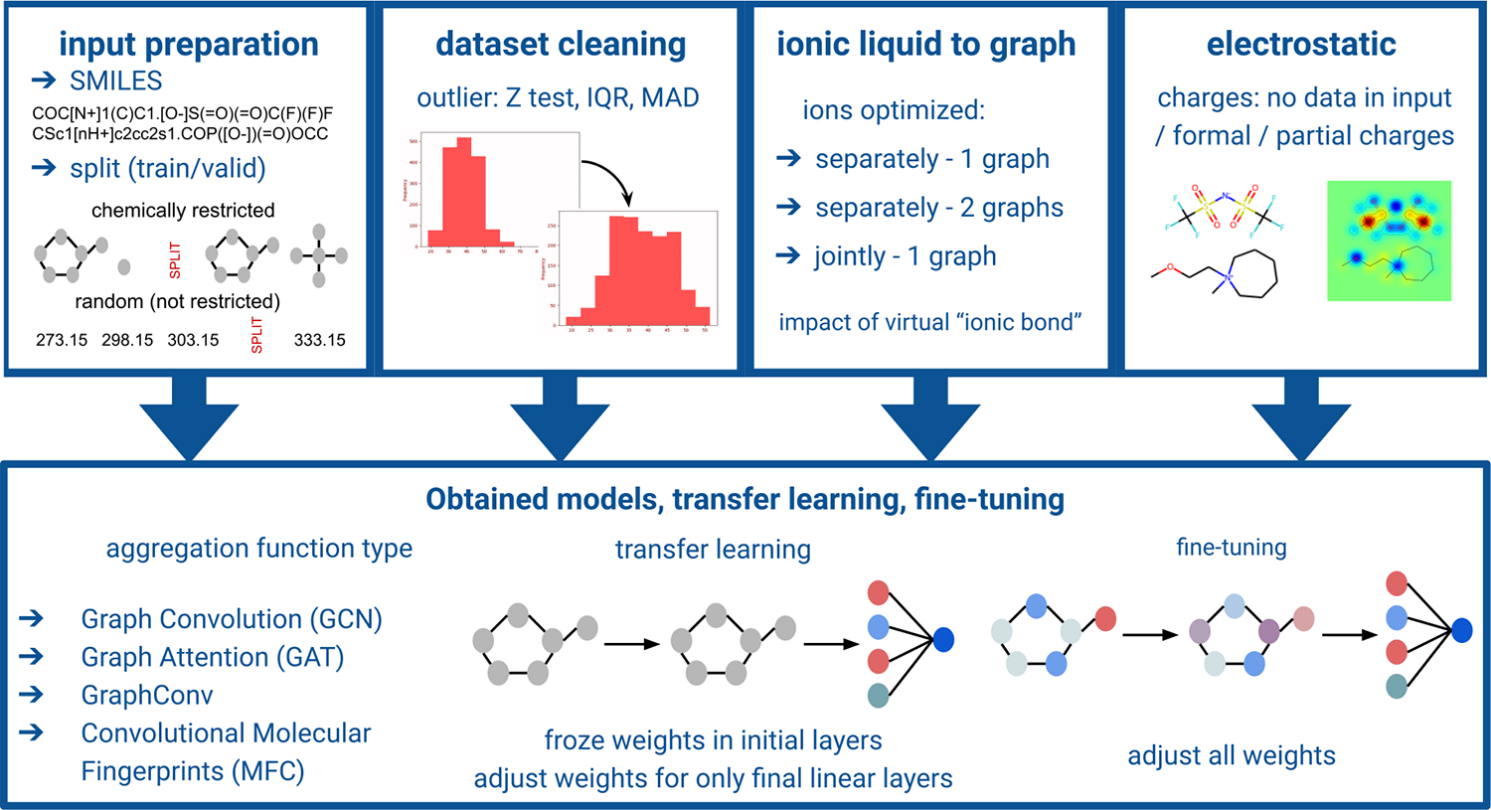
Overview of factors and methods investigated in the study.

Figure 1 shows how studied problems combine into a systematic study on GNNs application for molecular property prediction of ILs. First, input preparation and data set cleaning were discussed. Special attention was paid to modeling using an expertise-based cleaned data set that contains data of slightly lower reliability yet published in the literature on the topic. Moreover, different ways in which IL, as a pair of a cation and an anion, might be encoded into a graph were investigated. In addition, a study of the importance of electrostatic information for GNNs was made. Furthermore, the impact of the structure optimization tool, as provided in functions of Open Babel^30^ and RDKit,^31^ on GNN performance was checked. Finally, the models’ performance with tested possibilities on transfer learning and fine-tuning was discussed.

### Data Sets

In ML, the data set is a crucial component for building accurate models since a high-quality and diverse data set provides the necessary information for the model to generalize well to new data.

In this study, we used three data sets provided in the series of articles by Paduszyński and containing information on the density (ca. 40,000 points),^17^ viscosity (ca. 20,000 points),^18^ and surface tension^32^ (ca. 6000 points) of ILs. Since the databases are relatively up-to-date and there are no more extensive studies in the field, these databases could be treated as benchmarking in the field of structure–property modeling of ILs. Analysis of the chemical diversity of the data set, types, and numbers of different cations and anions, temperature and pressure ranges, ranges of property values, units, and experimental uncertainty is available in publications introducing databases.

Further in the text, there are references to the preprocessing of databases done by Paduszyski and his terminology referring to preparing the database for modeling. Therefore, the term “clean” data set refers to a data set after preprocessing suggested in the original article. On the other hand, the term “raw” data set represents a data set collected at original publications without any critical assessment.

### Outlier Detection

Outliers are data points that are significantly different from the majority of the data in an ML model. These data points might have a big impact on the model’s performance, as they can skew the results and lead to inaccurate predictions. Since there might be outliers in the data set, its detection using several algorithms was studied. Algorithms used for that purpose were based on *Z* score, interquartile range (IQR), or median absolute deviation (MAD).^33^ The *Z* score technique calculates the scores for each observation based on the mean and standard deviation of all observations. Extremely high or low *Z* scores, typically above 3 or below −3, denote outliers. The IQR outlier detection method involves calculating the distance between the first and third quartiles (Q1 and Q3) in the data. Observations more than 1.5 times the IQR below Q1 or 1.5 times the IQR above Q3 are considered outliers. The IQR and corresponding limits are resistant to outliers, making the method suitable for small data sets. The median absolute deviation technique uses the MAD, the median of the absolute deviations from the data’s median, as a measure of variance. Observations exceeding 2.5–3.5 times the MAD from the median are classified as outliers. Like the IQR, MAD is robust against outliers, making it a preferred method for analyzing skewed data. All data cleaning was performed using Pandas and Numpy libraries for Python.

### Basic Concepts of GNNs

In GNNs, graphs are used as data structures to represent complex relationships between entities. Using graphs as a data structure, GNNs can capture the dependencies and interactions between entities, making them well-suited for tasks such as molecular property modeling.^25^ Formally, graph *G* = ({*V*}, {*E*}) can be denoted as a pair of vertices *V* (nodes) and edges *E*.

Learning in GNNs is a process of updating information stored in graph structure while maintaining its integrity.^34^ Since neural network learning is basically an optimization process whose goal is to minimize model error, it is done using back-propagation, during which new model weights θ are obtained in accordance with the change of loss function with model weights change *∂L*/*∂*θ multiplied by learning rate depicting how conservatively weights are updated:
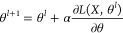


Thus, it could be stated that information is passed from one node to another node in a graph, and therefore, this neural-message-passing schema is a more generalized version of matrix convolution. Consequently, the graph structure is preserved, and there is no need to normalize molecules of different sizes to fit into one unified matrix for all samples' matrix sizes. One of the techniques used in GNNs, namely, neural message passing,^35^ is mathematically interpreted as a more generalized convolution. It is performed according to the formula:
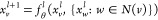


where:

1. *l* represents the iteration step.
2. *N*(*v*) represents the set of nodes connected with *v*.
3. *x_v_* represents the a vector of features of a node *v*.
4. *f* represents the aggregation function taking into account features of node *v* and all its surroundings.

One of the simplest aggregation function is the weighted average (which will be further denoted as GCN in accordance with the nomenclature used in Pytorch-geometric implementation):
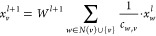
where *W*^l+1^ is the weights adjusted during model training (depicting how features are summed), and *c*_*w*,*v*_ is a normalization factor.

During this process, information is shared from one node through its connection by edges to other nodes. Therefore, each node represents the average information from itself and its surroundings. Since averaging the information might be obtained in several ways, there are many possible functions executing that process. In this study, four of the samples were tested. Types of graph networks studied in this work are

1. Graph convolutional networks (GCN)^36^—GCNs aggregate the neighbor feature information from each node so it can be weighted and passed to neighboring nodes.
2. Graph attention networks (GAT)^37^—information is not just simply weighted like in GCNs but rather calculated by a normalized attention function taking into account embeddings of nodes between which message passing occurs.
3. Convolutional GNNs without neighborhood normalization (k-GC)^38^—the network has an alternative to GCN formulation of convolution operation and allows hierarchical architecture and, therefore, is learning features of subgraphs of an input graph.
4. Convolutional networks learning molecular fingerprints (MFCs)^39^—traditional circular fingerprints were redefined by replacing hashing, indexing, and canonicalization with differentiable substitutes, leading to a novel type of graph convolution.

After the neural message passes, the readout phase occurs. During the readout phase, the pertinent structural data associated with each node are subsequently amalgamated into a continuous vector depiction of the graph. This amalgamation of the hidden states of individual nodes is achieved through utilization of a pooling function, such as summation. The resultant vector assumes the role of an input for a subsequent multilayer perceptron.

Transfer learning, a fundamental concept in deep learning, is gaining prominence in the domain of cheminformatics. This approach leverages pretrained neural network models, which have been initially developed on a vast data set for a related task, and fine-tunes them for the specific property of interest. Transfer learning enables the model to adapt its knowledge and representation of chemical structures from one domain to another, substantially reducing the need for extensive labeled data and accelerating the development of robust predictive models. The pretraining phase involves training a GNN model on a larger and more diverse chemical data set, where the network learns mostly how to encode general molecular features and relationships between atoms and bonds. Subsequently, the model is fine-tuned on a smaller data set for which less experimental data are available, allowing it to specialize in predicting this physicochemical property. Fine-tuning involves modifying the model’s parameters through additional training while preserving the knowledge gained during pretraining. The fine-tuning process seeks to update the model parameters θ, taking their initial values from the model trained in the first step rather than random values. Therefore, initial model parameters θ already represent some knowledge. This two-step process harnesses the generalization capabilities of the pretrained model and tailors it to the nuances of the chemistry of certain properties, enhancing its predictive accuracy. Transfer learning in this manner enhances the model’s capacity to extrapolate valuable insights from limited, specialized data and demonstrates its potential for the advancement of molecular property prediction in the field of cheminformatics. Transfer learning is extensively examined in the field of GNNs.^28,29^

### Modeling and Neural Network Architecture

In this study, the architecture of the proposed network consisted of 4 graph convolution layers with 128, 256, 256, and 128 neurons followed by 2 linear (fully connected) layers. The nonlinear activation function ReLU was incorporated. The first linear layer contained 256 neurons plus one neuron per condition, and the second layer contained 128 neurons. A dropout of 0.2 after convolutional layers was applied. Additionally, to drop out before the final layer, batch normalization was applied. During training, mean squared error as a loss function was minimized with Adam as the optimizer. The learning rate was set to 0.001, with the Cosine Annealing scheduler restarting every 40 epochs. The learning was performed for 300 epochs, which were sufficient for the GNNs to converge in all studied scenarios. Neural network architecture was obtained by reducing architecture from other studies in the field^24^ until metrics were significantly lowered to obtain networks that do not overfit the data. All operations on neural networks were performed using PyTorch^40^ and PyTorch Geometric^41^ libraries for Python.

### Chemical Representation of Molecules

**Figure 2:**
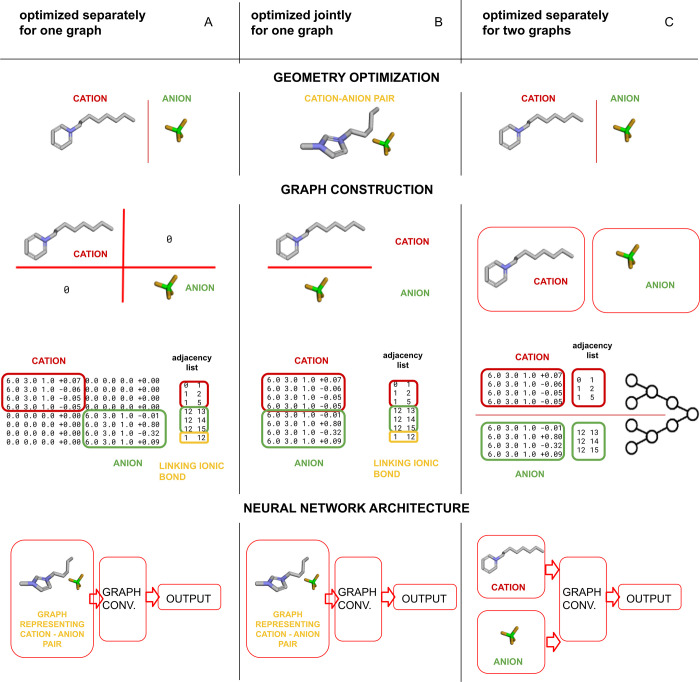
(A–C) Different ways of providing structural information on ILs for GNNs.

Molecules might be interpreted as graphs by representing each atom as a node and each bond as an edge connecting two nodes. For this purpose, a proper molecular structure is needed. Even though bond angles or lengths are not used as features in this study, molecular geometry affects, for example, partial charge calculations. Representation as graphs of substances composed of two molecules (species), such as ILs, should be more heavily investigated. There were three options examined differing in molecular geometry optimization schemas, which subsequently lead to different network configurations. In the first scenario (Figure 2A), the cation and anion molecular structures were optimized separately (treated as separate molecular graphs) and, subsequently, were represented as one matrix. This matrix combining occurred in a manner in which the cation graph was in the left upper corner, the anion graph was in the right lower corner, and the rest of the matrix was filled with null values. In the second scenario (Figure 2B), ILs as a cation–anion pair were treated as a single graph. Molecular structures were optimized in total as if they were one molecule. However, due to consistency, atoms from the cation are listed above those from the anion. In the third one (Figure 2C), two separate graph convolutions (separately for cation and anion) were performed in parallel, and matrix concatenation was not applicable. In that case, the cation and anion vectors were joined before the final linear fully connected layers. Scenarios A^24^ and C^26^ were used previously without an in-depth analysis of reasons favoring their usage. In this work, a novel factor, i.e., the impact of virtual ionic bond insertion (as a factor that eases the passage of information in the graph), was studied for these two scenarios. The ionic bond is represented as an additional edge, with weight −1, added to the end of the adjacency list (as represented in yellow and text “linking ionic bond” in Figure 2). Virtual ionic bonds are applicable only in scenarios A and B. An additional edge representing the virtual ionic bond was added in such a manner that the first atom in the cation molecule is linked to the last atom in the anion molecule. The exact order of linking of the atoms is arbitrary. However, it is not expected to impact graph convolution significantly. It is worth noting that every possible option will be somehow misleading because real liquid systems are dynamic. It is also expected that between scenarios A and B there should be a significant difference in the value of charges associated with atoms. This is because ion screening during optimization should vastly affect that parameter. All the scenarios are presented visually in Figure 2. The chemical structures and matrices presented in Figure 2 depict the main concept of each examined hypothesis in an illustrative manner and do not directly correspond to specific matrices or graphs employed in the study.

For proper graph creation, optimization of chemical geometry and proper molecular feature calculation are essential. Each entry in the database covers the name of IL, its SMILES textual representation, conditions (for density data set—temperature and pressure in which the experiment was conducted; for viscosity and surface tension data sets, only the temperature was provided), and the value of the physicochemical property. Then, SMILES was converted into a molecular object using the RDKit library for Python.^31^ Structures were optimized using Open Babel^30^ or RDKit^31^ using a conformational search strategy coupled with force field (FF) optimization. A key argument favoring a computationally determined FF is the uniformity and range of performance that parametrization confers against standard computationally expensive theoretical models.^42^

Regarding the way of transforming structural information into a graph, each atom in a molecule was treated as a node with the following properties: atomic number, number of hydrogens, hybridization, aromaticity, and charge. Chemical bonds were represented as edges connecting nodes with weights set according to the multiplicity and aromaticity of the corresponding chemical bond. To fully compress chemical information into a molecular graph, nearly all the readily available atom/bond-level properties were used.^43^ All the information was calculated using RDKit, except for partial charges, which were determined by both used chemical packages, namely, RDKit and Open Babel. Different methods exist for calculating partial charges, each with its advantages and disadvantages. The following are different types of partial charges incorporated as one of the input parameters for GNNs:

1. Gasteiger-Marsili^44^ sigma partial charges: This method assigns partial charges to atoms in a molecule based on the electronegativity of neighboring atoms and the number of bonds to those atoms. It is widely used in computational chemistry due to its speed and accuracy. However, the procedure was rather designed for neutral molecules than ions.
2. MMFF94 partial charges^42^: This method is part of the Merck molecular FF (MMFF) and is based on a combination of quantum chemical calculations and empirical fitting. It is known for its accuracy in predicting the properties of organic molecules. However, partial charge calculations strongly depend on the parametrization of the FF, which might not be the best choice for niche or novel classes of compounds.
3. Formal charges^45^: These are charges assigned to atoms in a molecule based on the number of valence electrons and the number of bonds to the atom. Formal charges are often used to identify important resonance structures and to explain the reactivity of a molecule. However, formal charges are not true partial charges but rather a way to assign charges to atoms in a molecule based on the number of valence electrons and bonds. They do not take into account the distribution of electron density in the molecule, so they can describe charge distribution inaccurately.
4. QTPIE partial charges^46^: This method takes into account charge transfer, polarization, and equilibration effects in a molecule. It is known for its accuracy in predicting the properties of small molecules and biomolecules. However, the method was designed for electrically neutral species.

### Data Set Splitting

The data set was divided into training, validation, and test sets. Special attention was paid to the test set. The proposed procedure was a little more complex when compared with a simple random split, but it is necessary to ensure that enough data are provided for training on clean and raw data sets. Therefore, the test set was drawn exclusively from the clean data set and, by assumption, covers 10% of entries (for the raw data set, the coverage was approximately 3%). Taking into account that the clean data set constituted 26% of the raw set, the obtained test set contained ca. 1% of unique liquids. Since data sets contain multiple data for one liquid (due to temperature dependency), it is beneficial to let the test set capture both structural and conditional relationships. Therefore, a test set was selected by the random selection of ILs together with associated data points (all measured for a given IL). The test set is selected by randomly choosing SMILES strings from the data set and assuring that they would not be used for training or validation of a neural network. It was assured that testing examples were selected only from the clean data set, so the model was not tested on samples whose solidness or comparability to other data was undermined. The same test set was used for both clean and raw data sets. The remaining parts of the data sets (clean and raw) were split in a proportion of 5:1 for validation and training purposes, so the overall proportion of sets was 75:15:10 for training, validation, and test sets for the clean data set, respectively.

Model performance was evaluated using metrics such as *R*^2^, RMSE, and MARE. *R*^2^ represents the proportion of the variance in the dependent variable explained by the independent variables in the model. RMSE (root mean squared error) is the measure of the differences between values predicted by a model and the actual observed values of the variable being predicted. Mean absolute relative error (MARE) is the average of the prediction errors divided by the true value of the property. For each mean, the metric value based on 4 runs (random seed values with 10 repetitions for each seed), as well as standard deviation, are provided.

## Results and Discussion

### Impact of Data Set Cleaning

Data cleaning is an important yet controversial step in the ML model-building process.^47^ The procedure protocols are strongly user-dependent and not interchangeable. In the series of articles sourcing the databases, cleaning was done via careful assessment of the comparability of data.^17,18,32^ In the first step of the database revision, data sets lacking essential sample information and experimental methods for property determination were excluded, and the data with the lowest declared water content were chosen as reference sets, although inconsistencies required manual inspection. Problematic data underwent auxiliary analyses and comparisons with similar data. In the second step, the remaining data sets were regressed using established equations on temperature dependence and other statistical methods with outliers detected and excluded iteratively based on statistical significance, as shown through Williams plots and leverage analysis. However, it leads to excluding many experimental findings, and modeling using only half or fewer of the data available in the literature might seem to be too strict an approach.

In the databases, there are about 40,000 points with experimental data for density, 20,000 for viscosity, and 6000 for surface tension. However, after a cleaning procedure applied by the author, only 58, 22, and 27% (respectively, density, viscosity, and surface tension) remain for modeling. Additionally, when comparing structural diversity in raw and clean data sets, it can be seen that only 16 or 24% (for viscosity and surface tension, respectively) of unique ILs in the raw data set are still present in the clean data set. Only for density, it can be stated that the raw and clean data sets cover similar chemical space since 93% of ILs present in the raw data set are also present in the clean one. Paduszyński used only the clean data sets for modeling and stated that modeling using the “raw” values produced incredibly low-grade and erroneous results.^18^ Therefore, it would be greatly beneficial to obtain a model that could utilize the data regarding whether they are immaculate. The adopted assumption obviously leads to the use of possibly large data sets, which enable the use of self-learning techniques, such as graph networks. This way would be possible to eliminate personal involvement by omitting the typical stage of clearing the collection of data of questionable quality and at the same time verifying the resistance of the methodology to the presence of questionable data.

In Table 1, model performance with different data set cleaning scenarios is presented. As a starting model setup, building schema using (i) separate optimization for one graph (compare Figure 2A) with (ii) formal charges and (iii) GCN convolution function was adopted. Model performance was evaluated on samples selected from the clean data set, so that model was tested on certainly reliable data. For each property, training on both raw and clean data sets was performed, and the best results are in bold. It could be seen that for the prediction of density (MAD detection, *R*^2^ = 0.955) and surface tension (no detection, *R*^2^ = 0.79), the most predictive models were obtained using raw data sets rather than models trained solely on a clean data set. In the case of viscosity, deterioration in the statistical parameters of the models was observed, which is discussed later. This observation might suggest that GNNs are able to handle mislabeled data efficiently. It is consistent with the previously reported fact that deep neural networks can generalize well even when some percentages of training data are not labeled correctly.^48^ However, this fact only expresses the observation of a model having equal performance when compared to the one trained on a clean data set. The metrics of the model should not exceed that value if only mislabeled data played a role. Therefore, obtaining even better performance by a model is governed by another factor. The observation might be explained by referring to chemical space covered in raw and clean data sets. The raw data set contains a much more structurally diverse set of ILs, resulting in GNNs better adapting to process structural information. Better adaptation is possible due to presenting to the network more diverse examples in the training set, leading to the optimization of convolutional layers to extract more general molecular features.

**Table 1:** Model Performance in Accordance with a Different Method of Dataset Cleaning

property	outliers detection method	raw data set		clean data set	
		data set size in % of raw data set (and its skewness)	RMSE/*R*^2^ ± standard dev. on the test set for a model trained on the data set	data set size in % of raw data set (and its skewness)	RMSE/*R*^2^ ± standard dev. on the test set for a model trained on the data set
surface tension	no detection	100% (0.52)	**3.19 ± 0.22/0.79 ± 0.05**	27% (0.44)	4.50 ± 0.29/0.60 ± 0.04
	*Z* score	99.3% (0.33)	3.91 ± 0.43/0.68 ± 0.17	26.9% (0.23)	4.96 ± 0.82/0.50 ± 0.18
	IQR	99% (0.29)	3.98 ± 0.50/0.66 ± 0.18	26.8% (0.22)	4.93 ± 0.93/0.51 ± 0.17
	MAD	96.7% (0.09)	4.17 ± 1.07/0.60 ± 0.15	26.3% (0.03)	5.02 ± 0.65/0.43 ± 0.07
viscosity	no detection	100% (39)	19.58 ± 1.88/0.55 ± 0.08	22% (16)	15.80 ± 1.92/0.70 ± 0.05
	*Z* score	99.3% (6.9)	20.53 ± 1.48/0.50 ± 0.09	22% (4.9)	15.90 ± 2.28/0.70 ± 0.08
	IQR	86.2% (1.8)	19.18 ± 1.50/0.56 ± 0.07	19.2% (1.5)	**16.18 ± 2.20/0.69 ± 0.06**
	MAD	76.2% (1.1)	19.75 ± 2.50/0.54 ± 0.10	17.4% (1.0)	16.01 ± 1.88/0.69 ± 0.05
density	no detection	100% (0.34)	75.77 ± 31.2/0.77 ± 0.14	58% (0.27)	42.59 ± 15.4/0.93 ± 0.06
	*Z* score	99.6% (0.13)	57.88 ± 23.4/0.87 ± 0.13	57.8% (0.10)	42.23 ± 13.9/0.93 ± 0.06
	IQR	99.7% (0.13)	66.63 ± 16.7/0.85 ± 0.08	57.9% (0.11)	36.24 ± 2.25/0.92 ± 0.07
	MAD	98.5% (0.01)	**34.76 ± 8.30/0.96 ± 0.02**	57.4% (0.02)	41.67 ± 13.1/0.93 ± 0.05

On the other hand, the results obtained for viscosity indicate that the quality of the input data must not be below a certain level. Undoubtedly, in this case, removing outliers from the data set to the level of 76% of the raw set was insufficient (the clean set contains only 17% of the raw data set). Therefore, careful preprocessing remains an issue, especially when a large amount of outliers is present, particularly in combination with the range of property value variation, which, in the case of viscosity, is extremely high and amounts to 7 orders of magnitude.

The results described earlier show that often there is no need to perform tedious cleaning as was needed for the classical group contribution method. Moreover, one does not need to predefine chemical groups that should contribute to the value of the physicochemical property, which is an additional benefit, making model creation even simpler. Nevertheless, the decision on the method of initial data preparation and its range should, in each case, be the result of knowledge of the specificity of the target parameter.

**Figure 3:**
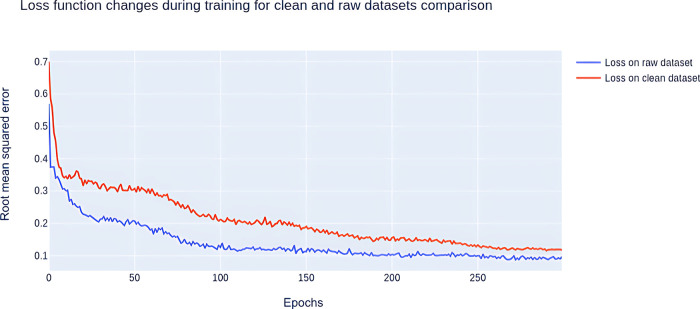
Loss function changes during training for clean and raw data set comparison.

Figure 3 shows how the loss function changes during the learning process by the example of a model for predicting density. The curve obtained for a clean data set seems to have three different slopes. In the first stage (approximately the first 20 epochs), the expected rapid decrease of loss due to adjusting initially random weights to the data takes place. In the second stage (epochs 20–100), slower yet fast loss minimization is observed. In the final stage, loss changes significantly, but converging to a minimum occurs slowly. In the case of a loss curve for a raw data set, the first two stages are similar to what is observed in the case of the curve for a clean set. However, after approximately 100 epochs, the model has almost converged to the minimum. This might suggest that using raw data sets not only improves performance but also decreases the number of epochs needed to reach convergence and might decrease training time.

From the point of view of building similar structure–property models on ILs with GNNs, it is worth noting that solely for prediction density and on the raw data set, one is able to overreach the value of *R*^2^ of 0.90. It might imply that the amount of data needed for that algorithm is exceptionally high. Based on the earlier discussion, the raw data set was selected as the default option in modeling, except for a highly skewed one (viscosity) for which a clean data set was selected.

### Impact of the Outlier Detection Technique

The results presented in Table 1 indicate that the impact of the outlier elimination method depends on both the type of property being modeled and the preliminary analysis of the collected data. It can also be assumed that the size of the analyzed set is also an important factor. In the case of models obtained for density (initially ca. 40,000 points), it can be seen that the extensive limitation of the set size at the curation stage (∼40% reduction) makes the outliers search stage redundant. The quality of predictions for models calculated using a clean data set does not differ significantly regardless of the computational technique used (0.92 < *R*^2^ < 0.933). On the other hand, a significant, user-assisted reduction in the training set may negatively affect the GNN learning process: in the analyzed case, only a slight correction of the raw set using the MAD technique (1.5% reduction) allowed obtaining a model with the highest predictive ability, described as *R*^2^ = 0.955. It turns out that the critical value in this case is the size of the training data set.

Somehow, the opposite case is the results obtained for viscosity (initially ca. 20,000). As already mentioned, this parameter is very specific from the point of view of predictive techniques because it is characterized by the largest range of variability. It is also important that this parameter be very sensitive to the quality (purity) of the liquid used in viscosity measurements. This last factor was the reason for the extremely large set reduction, which for the clean set meant the elimination of 83% of the output data. The obtained effect is partially similar to that observed for density: the obtained *R*^2^ values for all models trained on a clean data set are practically the same (0.688 < *R*^2^ < 0.695) for each of the outlier detection techniques used. Differences appear when comparing the results obtained using the raw data set. For viscosity, all trained models have lower predictive ability than those found using a clean data set. The quality of the model is also more dependent on the technique of indicating outliers: the difference between the lowest (*Z* score) and the highest (IQR) *R*^2^ is approximately 10%. It seems that the key is the relationship between the skewness of the distribution of parameter values and the range of its variability.

**Figure 4:**
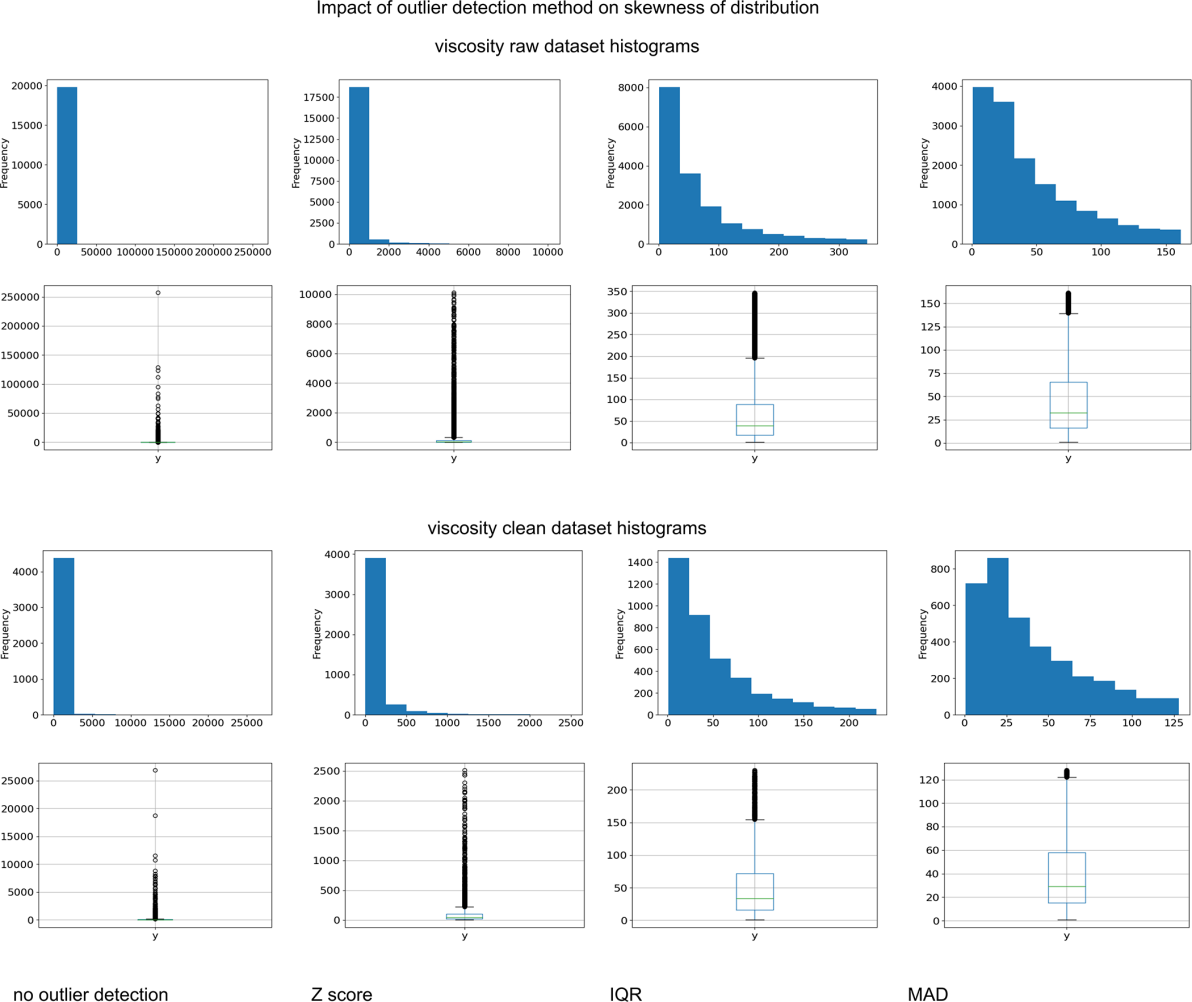
Outlier detection impact on distributions for viscosity raw and clean data sets.

It is the case for viscosity when the skew of the data set is as high as 39 without removing any outliers and 1.8 if choosing the IQR. Even when MAD was applied, skew is about 1.0, which is still quite high and might impact the performance of a neural network. However, it should be noted that the highest number of data is excluded in all cases while using the MAD detection method. Thus, data reduction reaches one-fourth of the output set and seems to be overdone, as it lowers the *R*^2^ value compared to the IQR model. Distribution changes over different outlier detection techniques are listed in Figure 4. It can be observed that the difference between the minimum and maximum values at distribution is 7 orders of magnitude. Moreover, the mean and median differ by 1 order of magnitude, which is an extreme example of an outlier impact. A similar situation occurs for the clean data set; however, the range of viscosity variability for each clean data set is several times smaller than for the raw set, which seems to be a decisive factor in this case.

Surface tension (initially ca. 6000) seems to be an intermediate case in terms of the importance of indicating outliers. First, it can be noticed that the data curation stage, with a large range of almost 75% reduction of the data (close to viscosity) does not ensure a significantly high quality of the trained models (*R*^2^ < 0.6). Moreover, all models found using clean data sets have significantly lower predictive ability compared with models obtained using raw data sets. It can even be noticed that a further, even slight, reduction in the amount of training data significantly worsens the quality of the models. For the most restrictive technique, the MAD *R*^2^ is only 0.43 compared with the value of 0.593 obtained using the intact clean data set. Interestingly, a similar effect can be seen for the raw data set, where the best predictive ability, *R*^2^ = 0.79, was obtained, as well as for the intact raw data set. The observations above clearly indicate that the factor limiting the quality of predictive models in the case of surface tension is the size of the training set, which is at the limit of the applicability of GNNs.

Finally, for further research, it was decided to use data sets with the highest training abilities for graph networks for each of the analyzed parameters (bold in Table 1).

### Different Splitting Scenarios

Mainly, two splitting scenarios into training and validation sets could be established. However, since this work focuses on structural information on ILs, the test set is treated entirely separately and covers ILs excluded from training and validation sets. Since temperature (and pressure) are also important inputs for a model, splitting can ensure that chemicals are (unrestricted) or are not (chemically restricted) present simultaneously in both sets. In the first case, neural network information about a whole temperature and pressure dependence of a property is given. That is not the case in the second case, of unrestricted split. In that situation, it is possible that just some data points (e.g., measurement in one exact temperature) are in a validation set, while some others (also measured for that compound) are represented in a training set.

A comparison of the two approaches was studied by the example of the surface tension data set since it is the smallest and contains the smallest chemical variety while guaranteeing the highest variation with temperature.

Performance in training sets is similar for chemically restricted splitting, and random one *R*^2^ on a train set is 0.84 and 0.83, respectively. However, performance in validation and test sets is significantly higher for random splitting scenarios. In the case of a validation split, the difference is about 20% (*R*^2^ = 0.83 for random split and *R*^2^ = 0.65 for restricted). Model performance for a test set on unseen structures is also significantly higher: the value of *R*^2^, while using the restricted split is about 0.72 compared to 0.79 for splitting, which ignores chemical information.

It can be concluded that if GNNs were trained on the full temperature range, it concentrates too much on properly predicting the temperature dependence rather than structural information. Even though temperature dependence is important, a model should leverage between structural input and conditions. That is obtained when using the split ignoring the chemical scaffold. Based on the findings, it was decided to use a random split in further analysis.

### Impact of Electrostatic Information on GNN Performance

ILs are compounds composed only of ions, and electrostatic information is a known factor of inconsistency between molecular simulations and experiments.^49^ Therefore, it would be interesting to study how a novel GNN algorithm deals with that kind of input information for predicting the properties of ILs. Model performance without considering partial charges is also investigated since some authors who use GNNs for structure–property modeling of simpler compounds do not use partial charges as part of their input.^50^

In modeling, charges were included for systems where the cation and anion were optimized separately and jointly, using 5 (no charge, formal charges, and Gasteiger-Marsili, MMFF94, QTPIE partial charges) different methods of calculating charges, which leads to 10 in total. Results are listed in Table 2. The geometry of a compound that is composed of cation and anion can be optimized in two scenarios—both ions simultaneously or separately (so without information about counterion). It can be seen that the impact of how an IL molecule was optimized is more important than the way of calculating charges. It might be the case since partial charges differ according to how atoms are oriented in space. The impact of screening the charge by other atoms is, therefore, greater than the estimation itself. Therefore, charge distribution seems to be more decisive for GNNs than the exact value of the partial charge itself. This statement is supported by the observed fact of good performance even while providing only formal charges since, in convolutional layers, the information is somehow distributed to nodes near the node with the assigned formal charges.

**Table 2:** Model Metrics with Different Ways of Calculating Partial Charges

charge representation	ions converted to one graph with separately optimized geometryRMSE/*R*^2^ (test set) ± standard dev.	ions converted to one graph with jointly optimized geometryRMSE/*R*^2^ (test set) ± standard dev.
no charges	3.23 ± 0.29/0.79 ± 0.06	3.48 ± 0.57/0.74 ± 0.11
formal charges	3.19 ± 0.22/0.79 ± 0.05	3.40 ± 0.39/0.76 ± 0.07
Gasteiger charges	3.28 ± 0.18/0.78 ± 0.04	3.54 ± 0.58/0.74 ± 0.11
MMFF94 charges	3.32 ± 0.13/0.78 ± 0.04	3.60 ± 0.42/0.73 ± 0.08
QTPIE charges	3.39 ± 0.28/0.77 ± 0.05	3.72 ± 0.46/0.72 ± 0.09

Studying more in-depth models trained with ions optimized separately, it can be observed that additional information about partial charges does not improve model quality significantly. Even a model that is not informed about charges in the system performs similarly to electronegativity-based (Gasteiger) or FF-based (MMFF) methods. This might suggest that GNNs, on their own, reproduced the information on charge distribution based on the atoms’ connectivity. However, introducing this information might lead to a decrease in the standard deviation of the predictions, as seen in a formal charge scenario.

Moreover, even for all studied methods, *R*^2^ is still about 0.70, which denotes that differences between the methods of calculating partial charges do not impact GNN performance by more than 5%. Therefore, it might be concluded that rough information on charge distribution is more important to obtaining a well-performing network than precise parameter-based or FF-based estimation.

Finally, it is worth mentioning that there is generally a slightly higher standard deviation observed for a model trained with no information about charges when compared to other scenarios. This might suggest that the model is less stable in prediction if it has to deduce electrostatic information solely from structural data.

For further analysis, formal charges were used in modeling due to their simplicity and promotion of a model with a low standard deviation. However, issues regarding geometry optimization strategies seemed to arise, and further analysis of the problem was performed in the following paragraphs.

### Comparison of IL Structure-to-Graph Conversion Methods

IL, since they arise from a combination of two separate molecules, is quite complex chemically. In consequence, there are different ways of transforming into a graph. The analysis was performed based on previous findings on ways of optimizing the structure of ILs and is presented in Table 3. Results are obtained, similarly as in Table 2, for the data set on surface tension.

**Table 3:** Different Ways of Transforming IL Structural Information into a Graph

engine	method		
	separately optimized geometry for one graph (*R*^2^ train/validation/test ± standard dev.)	jointly optimized geometry for one graph (*R*^2^ train/validation/test ± standard dev.)	separately optimized geometry for two graphs (*R*^2^ train/validation/test ± standard dev.)
Open Babel	with an ionic bond: 0.85 ± 0.02/0.84 ± 0.01/0.78 ± 0.05	with an ionic bond: 0.85 ± 0.01/0.84 ± 0.01/0.73 ± 0.10	separate pooling: 0.84 ± 0.02/0.84 ± 0.02/0.75 ± 0.16
	without ionic bond: 0.84 ± 0.02/0.84 ± 0.01/0.79 ± 0.05	without ionic bond: 0.86 ± 0.01/0.85 ± 0.01/0.76 ± 0.07	join pooling: 0.85 ± 0.01/0.85 ± 0.01/0.72 ± 0.13
RDKit	with an ionic bond: 0.85 ± 0.02/0.84 ± 0.03/0.78 ± 0.06	with an ionic bond: 0.86 ± 0.01/0.85 ± 0.01/0.75 ± 0.09	separate pooling: 0.84 ± 0.02/0.83 ± 0.03/0.75 ± 0.06
	without ionic bond: 0.84 ± 0.02/0.84 ± 0.01/0.78 ± 0.05	without ionic bond: 0.85 ± 0.01/0.84 ± 0.01/0.75 ± 0.09	join pooling: 0.84 ± 0.02/0.84 ± 0.01/0.74 ± 0.07

The engine used to optimize the molecular structure of ILs did not play an important role. Differences between studied scenarios of transforming IL into a graph were significantly larger than those caused by using different engines. However, Open Babel performed significantly better while dealing with structures. While using RDKit for the studied property, there were optimization problems for 20 of 279 ions. Even though RDKit was successfully applied in some previous work in the field of predicting ILs’ properties using GNNs,^26^ in the studies on the property under investigation, it failed to propose an initial 3D structure. On the contrary, Open Babel optimized geometry for all of the ions in the data set. Therefore, Open Babel, as a more robust tool, was selected for further analysis. It can be observed that the best model performance was obtained when IL molecules were treated as one graph but with separately optimized ions. Therefore, ILs might be treated as a mixture with some open space for including interactions between ions that are reflected in the convolutional layers.

Studies on the impact of adding a virtual ionic bond do not lead to consistent conclusions. In the case when separately optimized structures were provided to the GNN model, no difference was observed. However, when studying systems that were optimized jointly, the addition of that edge slightly improves the model quality. This leads to the further observation that dual molecular systems are not well described as a simple one molecule with structures of cation and anion optimized jointly.

All findings lead to the conclusion that ILs cannot be treated as simple organic molecules like in previously reported GNN models. Thus, it was decided to use structures optimized separately for one graph with a fictitious ionic bond in further analysis.

### Impact of Convolution Layer Type on GNN Performance

Finally, some experiments regarding the type of convolutional layer were performed. It was observed that both the GCN network and GAT network performed equally well on the test set, with *R*^2^ close to 0.79. Even though metrics in both cases are quite similar, there is a significant difference in standard deviations on the test set, as shown in Table 4. Results are obtained, similarly as in Tables 2 and 3, for the data set on surface tension.

**Table 4:** Comparison of GNN Model Performance According to Convolutional Function Type

convolution function	*R*^2^ (train set) ± standard dev.	*R*^2^ (validation set) ± standard dev.	*R*^2^ (test set) ± standard dev.
graph convolutional networks (GCNs)	0.84 ± 0.02	0.84 ± 0.01	0.79 ± 0.05
graph attention networks (GAT)	0.85 ± 0.01	0.84 ± 0.02	0.78 ± 0.07
convolutional GNNs (k-GC)	0.85 ± 0.03	0.84 ± 0.02	0.51 ± 0.21
convolutional networks learning molecular fingerprint (MFC)	0.91 ± 0.02	0.89 ± 0.02	<0

Moreover, another tested approach involved another implementation of graph convolution easing higher-order graph operations (k-GC), which resulted in a significantly lower *R*^2^ = 0.51. It might be stated that probably substructures that should be extracted in that procedure are not really as important for GNN prediction. Finally, the MFC network was tested since it was designed specifically for handling chemical structural data by reproducing molecular fingerprints. Unfortunately, the performance was not satisfactory. Even though performance in the training set for the property data is high (0.91 vs 0.84 in classical graph convolution), performance on the test set is poor (*R*^2^ < 0). It leads to the conclusion that this type of convolution layer is concentrated too much on structural features and generalizes poorly for novel compounds. Therefore, it was decided to use the simplest convolutional function in further analysis.

### Transfer Learning and Fine-Tuning Possibilities

It is a well-established fact that neural network architecture with weights might be transferred from one task to the other.^51^ However, transfer learning in GNNs is relatively poorly studied.^52^ Therefore, it might be interesting to investigate how well knowledge (in terms of weights) might be transferred from the neural network for predicting density (because its performance is sufficiently good) to other studied tasks. Two main scenarios were tested, namely, fine-tuning (updating all weights during training for novel tasks) and transfer learning (updating only final linear layers), as presented in Table 5.

**Table 5:** Summary of the Obtained Models, Transfer Learning, and Fine-Tuning Evaluation

pretraining neural network	model performance for predicting	*R*^2^ (train/valid/test ± standard dev.)	RMSE (train/valid/test ± standard dev.)	MARE (train/valid/test ± standard dev.)
—	density	0.97 ± 0.01/0.97 ± 0.00/0.97 ± 0.01	26.9 ± 0.88/28.5 ± 1.21/30.7 ± 1.88	0.016 ± 0.001/0.017 ± 0.001/0.019 ± 0.001
	viscosity (clean)	0.75 ± 0.04/0.74 ± 0.03/0.69 ± 0.06	15.56 ± 1.29/15.70 ± 1.02/16.18 ± 2.20	0.387 ± 0.078/0.369 ± 0.092/0.435 ± 0.074
	surface tension	0.84 ± 0.02/0.84 ± 0.01/0.79 ± 0.05	3.68 ± 0.16/3.75 ± 0.16/3.19 ± 0.22	0.070 ± 0.003/0.071 ± 0.002/0.068 ± 0.004
density (fine-tuning)	viscosity (clean)	0.85 ± 0.02/0.83 ± 0.03/0.61 ± 0.09	18.8 ± 0.54/20.1 ± 1.84/28.6 ± 2.00	0.379 ± 0.035/0.386 ± 0.100/0.737 ± 0.234
	surface tension	0.92 ± 0.01/0.90 ± 0.02/0.71 ± 0.09	2.65 ± 0.09/2.90 ± 0.30/3.80 ± 0.77	0.050 ± 0.000/0.053 ± 0.002/0.074 ± 0.012
density (transfer learning)	viscosity (clean)	0.68 ± 0.02/0.65 ± 0.03/0.54 ± 0.13	28.2 ± 0.38/29.0 ± 1.46/30.7 ± 4.42	0.595 ± 0.05/0.606 ± 0.13/1.0 ± 0.37
	surface tension	0.80 ± 0.01/0.80 ± 0.02/0.67 ± 0.10	4.12 ± 0.08/4.18 ± 0.20/4.02 ± 0.45	0.081 ± 0.001/0.082 ± 0.002/0.086 ± 0.008

Based on the findings presented earlier, for final modeling, it was decided to use IL geometry optimized separately for each ion and combined into one graph without fictitious linking ionic bonds augmented with information on formal charges. The raw data set was used, except for building a model predicting viscosity when a clean data set was used. In all cases, the outlier detection that performed the best for each property was incorporated. During training, random splits and simple GCN neural network architecture were used.

Fine-tuning and transfer learning lead to poorer results (than training solely on viscosity and surface tension data), therefore suggesting that structural features are not very transferable directly without any change from one task to the other. This is the case since, in the surface tension data set, there are ILs with side chains much larger than present in the density data set. Therefore, structural diversity is probably the limiting factor in that case. Overall, models obtained using pretraining have similar predictive performance on novel unseen compounds when compared to models without pretraining.

For predicting density in the original studies, databases *R*^2^ = 0.99, RMSE = 27.3, and MARE = 0.015. In the case of modeling viscosity, the best models were described by *R*^2^ = 0.84 and MARE = 0.377. The surface tension prediction model obtained *R*^2^ = 0.99, RMSE = 4.7, and MARE = 0.085. In comparison to those metrics, presented models seem to have lower performance than state of the art. However, the presented approach allows for using more experimental data with a simpler data-cleaning stage, has a simpler model-building procedure (due to no need to provide), and relies on testing the model on ILs previously unused during model training (which is not properly assured in the original studies).

A notable limitation of the study lies in the intrinsic complexities associated with the prediction of the physicochemical properties of ILs using graph neural networks. While the primary objective of the research is to elucidate how structural inputs should be processed to optimize neural network performance, several challenges have been encountered. The experimental database encompasses several hundred to approximately 2000 distinct ionic liquids, yielding up to 40,000 experimental data points. However, inherent issues such as variations in the temperature and pressure during data collection have presented substantial challenges. These variations introduce significant uncertainties in the measured properties, particularly in the case of viscosity, where differences spanning several orders of magnitude can be observed. Consequently, these uncertainties have the potential to impact the accuracy of the predictions, especially when attempting to capture the subtle nuances within the data. Furthermore, the models’ interpretability is hindered by the neural network architecture, particularly when incorporating temperature and pressure information, making it considerably more challenging to discern the underlying chemical insights driving the predictions.

Another limitation of the study pertains to the generalizability of the models to diverse ILs and physicochemical conditions. While the performance of the GNNs is commendable, it falls slightly short of state-of-the-art approaches. Nevertheless, it is crucial to acknowledge that the proposed model-building process offers distinct advantages. Notably, it eliminates the need for laborious feature engineering and preprocessing associated with traditional group contribution features or molecular descriptors. Moreover, the model demonstrates robustness in handling mislabeled data, particularly in the context of density and surface tension predictions. Despite these merits, the potential for improvement in predictive accuracy remains, and further research is warranted to address the limitations arising from the inherent experimental uncertainties. Additionally, the availability of computational resources, while generally accessible, could potentially be a constraint for researchers with limited access to high-performance GPUs, as the efficiency of training and evaluation can be influenced by hardware resources. Overall, while the study represents a significant step forward in predicting the physicochemical properties of ionic liquids using GNNs, these limitations underscore the necessity for ongoing research to enhance the robustness and applicability of the proposed approach.

## Summary

GNNs were shown to perform well in predicting the physicochemical properties of ILs.

The conducted analysis of the influence of factors influencing the quality of models has led to several interesting conclusions. First of all, GNNs turned out to be relatively resistant to the presence of data of uncertain quality in the training set. For density and surface tension, the performance of the models obtained was better when using the raw set than the cleaning one. Nevertheless, it should be emphasized that the indicated GNN capabilities have limitations. In the case of a data set with high skewness and high variability in the output, careful analysis of the data set is advisable. Thus, the tedious and time-consuming data-cleaning step might be reduced substantially or, in some cases, omitted. An interesting conclusion results from the evaluation of the charge representation methods. For ionic compounds, i.e., ILs, we have shown that the method of calculating the charges is not crucial for the performance of the model. To obtain satisfactory results, it is enough to use relatively simple calculation methods based on the comparison of the electronegativity differences between atoms. Important conclusions concern the method of transferring information about the ion pair to the graph, both in storing information about the cation–anion interactions and in transferring this information to the graph. The performed calculations clearly showed that this stage is of key importance for the quality of the models, where the optimal solution turned out to be the creation of one graph made of separately optimized ions connected by a virtual ionic bond. This work also showed that GNNs show high efficiency in the case of shifting between different modeled parameters. GNNs trained for density were able to model both viscosity and surface tension with great efficiency. At the same time, it has been shown that updating all weights during training for novel tasks (fine-tuning) provides better results than only fine-tuning (transfer learning).

## Accession Codes

The underlying code for this study is available and can be accessed via the link to the GitHub repository at: https://github.com/kbarn411/GNNs-and-ILs-properties.
